# Physicians’ Practices in Diabetic Nephropathy in Primary Healthcare Centers in Jazan City, Saudi Arabia, 2023

**DOI:** 10.3390/medicina60030372

**Published:** 2024-02-22

**Authors:** Amal J. Alfaifi, Ahmed Y. Abdaly, Bashaer M. Ghazwani, Ibrahim M. Gosadi

**Affiliations:** 1 Department of Family Medicine, Jazan Health Affairs, Ministry of Health, Jazan 82611, Saudi Arabia; ahmdabdly1@gmail.com (A.Y.A.); bashayer.33.b@gmail.com (B.M.G.); 2 Department of Family and Community Medicine, Faculty of Medicine, Jazan University, Jazan 82621, Saudi Arabia; gossady@hotmail.com

**Keywords:** practice, physician, diabetic nephropathy, Jazan City

## Abstract

*Background and Objectives*: Diabetes is one of the most common diseases dealt with by physicians in primary healthcare centers (PHCs). The disease is associated with macrovascular and microvascular complications, especially in those with long disease duration and uncontrolled diabetic nephropathy, which is one of the most common microvascular complications among diabetic patients. This investigation assessed the practices of physicians working at PHCs in terms of diabetic nephropathy screening, management, and referral. *Materials and Methods*: This study is a cross-sectional investigation targeting physicians working at PHCs in the Jazan region of Saudi Arabia between March and August of 2023. Data were collected via a self-administered questionnaire, which was distributed via online platforms. The questionnaire included sections measuring physicians’ demographic data and associated factors regarding training, the availability of resources, and practices in diabetic nephropathy, including screening, management, and referral. Chi-squared tests were used to assess associations between the practices of physicians and the measured demographics. *Result*: A total of 234 physicians participated in the investigation. The median age of the participants was 35 years. The adherence level of practice toward diabetic nephropathy according to American Diabetes Association (ADA) guidelines ranged from 40 points (the highest adherence level of participants) to 19 points (the lowest adherence level of participants), with a median of 33 points. Higher adherence levels were noted among physicians in Saudi Arabia, physicians with higher education levels, physicians specializing as family physicians or diabetologists, physicians who reported attending online and on-site training at diabetic centers, physicians who reported continuous access to urine and serum creatinine tests, and physicians who reported continuous access to the American Diabetes Association guidelines (*p* < 0.05). *Conclusions*: There are several factors associated with the level of adherence in diabetic nephropathy practice, such as physicians’ education level, specialty, training, and access to guidelines. The findings suggest the need for more training for PHC physicians in the care of patients affected by or at risk of diabetic nephropathy.

## 1. Introduction

The countries of the Arab Gulf Cooperation Council are considered to be situated in one of the regions with a high prevalence of diabetes. According to the latest International Diabetes Federation report, the frequency rate of diabetes in these countries including the Kingdom of Saudi Arabia, Bahrain, Qatar, Oman, Kuwait, and the United Arab Emirates, all of which have similar frequency rates of diabetes, ranges from 8% to 22% [1]. Diabetic nephropathy is the leading cause of end-stage renal disease (ESRD) [2] and one of the microvascular complications of diabetes (especially uncontrolled), with long-term complications. Early screening, management, and intervention make a difference in clinical care [3].

Almost one-third of the Saudi Arabian population with type 2 diabetes has diabetic nephropathy. In 2011, it was estimated that 42.5% of ESRD cases in Saudi Arabia were related to diabetes, and the cost of dialysis in the country amounted to SR 173,784 (US 46,332) per patient per year [4].

A cross-sectional study published in 2023 included patients with type 2 diabetes mellitus who visited the diabetes clinics of primary healthcare centers (PHCs) of two National Guard hospitals in the Eastern Province of Saudi Arabia. The study included 935 patients with type 2 diabetes (54.1% females and 45.9% males). The research found that microvascular complications in this region were common in patients with type 2 DM. The prevalence of microvascular complications in DM was 55.1%. The most typical complications that patients experienced were nephropathy (80.2%), retinopathy (32.7%), and neuropathy (8.4%) [5].

Diabetic nephropathy is the leading cause of end-stage renal disease. It is one of the common microvascular complications of diabetes. The earliest change triggered by metabolic factors is mainly hyperglycemia [4], and hypertension is considered one of the risk factors [6].

Most patients with diabetes receive follow-up care in primary healthcare centers. It is very important to detect complications early by screening to prevent deterioration and advanced complications. Diabetic nephropathy is usually asymptomatic, and symptoms do not develop until patients progress to late-stage grade IV–V chronic kidney disease. The usual screening of diabetic nephropathy uses the albumin/creatinine ratio, which is normal at <30 mg. Microalbuminuria is indicated between 30–300 mg, and >300 mg indicates macroalbuminuria [7]. Calculating the glomerular filtration rate (GFR) is very important in the annual screening of patients with diabetes to detect the stage of kidney disease [4].

The pattern of diabetic nephropathy among non-insulin-dependent diabetic patients in Saudi Arabia is similar to that in the Western world. The duration of diabetes and hypertension is an important risk factor for diabetic nephropathy, and there is a good correlation between nephropathy and the degree of proteinuria [8]. A cross-sectional study of diabetes in Lebanon found that the prevalence of microvascular complications was 33% among diabetic patients [9]. Therefore, the screening and early detection of diabetic kidney disease is very important in practice to prevent progression to a late stage and the need for dialysis. When most patients with diabetes receive follow-up care in PHCs and are asymptomatic for diabetic kidney disease, especially in the early stage, they should be screened to address the problem from the beginning to prevent complications, irreversible damage, and the need for dialysis.

It is important to assess the degree of applying the established guidelines pertaining to diabetic nephropathy in real-life clinical settings in a country that has a high prevalence of diabetes and, subsequently, a high risk of development of diabetic nephropathy. This emphasizes the importance of establishing screening for diabetic nephropathy as an innovative indicator for the management of patients with diabetes and the prevention of the development of complications. This indicates the importance of enhancing the effectiveness of screening services in primary healthcare settings to enable the detection of diabetic nephropathy cases in the early stages. According to the ADA, screening for diabetic nephropathy should be initiated immediately at the time of diagnosis in patients with type two diabetes. In clinical settings where laboratory infrastructure required for assessment of the value of urinary albumin excretion (UAE) is not available, which is the case in many primary healthcare settings, it is recommended to establish screening for diabetic nephropathy via semiquantitative dipstick measurement of albuminuria as an alternative and to receive follow-up care in the form of confirmatory diagnostic and clinical testing [7]. This study aimed to assess the screening, referral, and management practices of primary healthcare physicians in diabetic nephropathy and assessment of factors associated with practice adequacy.

## 2. Materials and Methods

### 2.1. Study Design and Settings

This study was a cross-sectional investigation conducted in the Jazan region in southwestern Saudi Arabia. This study targeted physicians working at PHCs in the region. Data collection was performed between March and August of 2023. Approval to conduct this study was granted by the Jazan Ethics Committee (number 2333). An electronic consent form was completed by the physicians who agreed to participate in the investigation. Data collection was anonymous and did not include any identification data.

### 2.2. Data Collection Tool

Data were collected via a self-administered questionnaire. The questionnaire was composed of three main components measuring the demographics of the physicians, the availability of facilities and services needed for the management of patients with diabetic nephropathy, and their reported practices when being visited by a patient suffering from or at risk of developing diabetic nephropathy. The content on practices in diabetic nephropathy was constructed by consulting relevant literature on diabetic nephropathy [7,10].

The validity and reliability of the developed questionnaire were ensured by performing the following steps. First, after selecting items relevant to assessing the physicians’ practices concerning diabetic nephropathy, a panel of experts was formed, comprising consultants in nephrology and family medicine. The panel was requested to complete a content validation form and provide a scale concerning adherence to diabetic guideline levels in diabetic nephropathy practice [11]. This was followed by an assessment of the face validity of the questionnaire using a sample of 10 male and female physicians to test the clarity of the questionnaire and the time needed to complete it. Finally, the reliability of the questionnaire was tested using Cronbach’s alpha, providing a value of 0.719.

### 2.3. Data Collection Process

After developing the questionnaire and testing its validity and reliability, the questionnaire was converted into an electronic format using Google Forms, and a web link was generated to facilitate access to the questionnaire. To obtain this study’s sample, administrative approval was secured to facilitate access to the physicians’ communication channels, including work-related WhatsApp groups, and distribution of the questionnaire web link via the relevant PHC administrations, thus completing the identification and engagement phases of data collection. Physicians who provided informed consent electronically were granted access to the questionnaire. Those who completed the questionnaire were designated as recruited participants, thus completing the final stage of the data collection process.

### 2.4. Data Analysis

Data analysis was performed using a statistical package for social science software (version 25). A descriptive analysis was conducted using frequency and proportions for binary and categorical data, while means, medians, standard deviations, and interquartile ranges were used to summarize continuous data according to their distribution. The descriptive analysis was followed up by inferential statistics to assess factors associated with knowledge and practice in diabetic nephropathy among the participating physicians.

The inferential analysis was initiated by estimating the level of practice. In this investigation, 14 items were used to measure the physicians’ levels of practice regarding diabetic nephropathy. Each item was given a score of 1 if the physician selected and practiced it correctly. The completed items were added to provide a practice score. The median value of the scores was utilized as a cut-off point to classify each physician as having lower or higher adherence to diabetic guideline levels in diabetic nephropathy practice.

The Chi-squared or Fisher’s Exact tests were used to assess differences in practice according to the measured physicians’ characteristics. To enable cross-tabulation, continuous variables, such as age, were classified into two groups according to the median. Furthermore, to avoid the presence of empty cells, specialties were grouped according to physicians who were general practitioners, family physicians, or diabetologists. Additionally, education level was grouped according to physicians with a master’s degree or lower and those who were board-certified or held a Ph.D. A value of *p* < 0.05 was designated as statistically significant for the applied statistical test.

## 3. Results

The total number of physicians who agreed to participate in this study was 234. The demographic characteristics of the recruited physicians are displayed in Table 1. The mean age of the physicians was 35 years, with a mean experience length of 7 years. The proportion of male physicians was higher than female physicians 59%, while more than half of the sample were Saudi Arabian physicians (58%). The majority of the participants were Arabic speakers (86%). When the physicians were asked about their specialty, nearly half were general practitioners; the remaining physicians were family physicians, and only one was a diabetologist. Upon asking the physicians about their education level, more than half held a bachelor’s degree, and only 33% were board-certified.

Table 2 summarizes the reported availability of training, services, and resources needed to provide healthcare for patients with or at risk of diabetic nephropathy, according to the recruited sample of physicians. When the participants were asked whether they had received training in diabetes management, half of the sample reported they had received training, either on-site or virtually, while 35 physicians reported they had not received training in diabetes management. Only 30% of the physicians indicated they had received clinical training at a diabetes center, and fewer than 10% reported receiving clinical training at a nephrology clinic.

Table 3 displays the availability of services and resources in primary healthcare centers concerning the management of diabetic nephropathy as reported by the recruited physicians. When the physicians were asked about the availability of resources concerning the care of patients with or at risk of diabetic nephropathy, only 41% indicated that glycated hemoglobin tests were always available at their centers. Similarly, 70% of the physicians reported that urine analysis tests were always available in their centers, while only 44% reported having serum creatinine tests continually available. The majority of the physicians reported access to the Wasfaty service (85%), which is a service provided by the Saudi Arabia Ministry of Health to facilitate medication prescription services.

When the physicians were asked whether they benefit from the consultation services of other clinics, only 22% reported that they always have access to consultations with a specialist, only 19% reported having access to consultations with a nutritionist, and only 30% reported having access to consultations with a health coach. Finally, when the physicians were asked if they have access to clinical guidelines for the management of patients with diabetic nephropathy, more than half of the sample reported having continuous access to the ADA guidelines, while only 15% reported having continuous access to kidney disease improving global outcomes (KDIGO).

Table 4 summarizes the practices of physicians regarding diabetic nephropathy, including screening, management, and referral. The reported frequency of screening for diabetic nephropathy was as follows: when the participants were assessed on the performance of routine screening of diabetic nephropathy, 181 physicians (77.4%) said they perform annual screening, which is a good practice according to the recommendations. The reported usual start of screening of diabetic nephropathy in type 1 diabetes was as follows: 122 physicians (52.1%) perform screening 5 years after diagnosis, which is good practice since type 1 has a mostly acute onset with the start of complication delay so screening (according to recommendations) starts 5 years after diagnosis.

For type 2 diabetes, 177 physicians (75.6%) start screening for diabetic nephropathy at diagnosis, which is correct practice according to the recommendations. Since patients have type 2 diabetes for a long time before diagnosis, the recommendation is to start initial screening at diagnosis, in contrast to type 1, in which screening starts after 5 years. Regarding the method used to screen for diabetic nephropathy, 200 physicians (85.5%) use the albumin-to-creatinine ratio, which is recommended by the guidelines as a measure for screening diabetic nephropathy. The ADA creatinine ratio guidelines can be summarized as follows: microalbuminuria is indicated between 30–300 mg, and >300 mg indicates macroalbuminuria; if there is a positive result of albumin, conduct a second test to confirm the positive test and also check for other factors that cause high urine albumin, such as heavy exercise and high levels of protein and some herbal medicines.

The number of visits is determined according to the result of the screening test; when the result of the albumin to creatinine ratio is 300 mg/g creatinine and GFR ≥ 90 mL/min/1.73, 51 (21.8%) physicians schedule two appointments per year, which indicates high adherence according to the recommendations. When the result of the initial screening albumin to creatinine ratio is 300 mg/g and GFR = 30–60 mL/min/1.73, 100 (42.7%) physicians provide two appointments per year, which is recommended by the guidelines.

The second part of the assessment was practice in management. Among patients with type 2 diabetes with chronic kidney disease with GFR > 25 mL/min/1.73 and urinary albumin of 300 mg/g creatinine, 137 (73.9%) physicians prescribe patients empagliflozin, which is a good practice among patients with diabetic kidney disease to prevent and delay the progression of the disease. In patients with type 2 diabetes, chronic kidney disease, and risk of cardiovascular disease, the contraindication is to use a sodium–glucose transport inhibitor; only 61 physicians (26.1%) use finerenone, which is recommended to prevent and delay progression in kidney disease if an SGLT2 inhibitor is contraindicated.

After starting patients with diabetes on angiotensin-converting enzyme inhibitor (ACEI) drugs, when the serum creatinine increases by <30% without volume depletion, 141 (60.3%) physicians continue the same medication, which is good practice according to the recommendations. Among patients with diabetic kidney disease on dialysis, 54 physicians prescribe 0.8 g/kg body weight/day, which is good practice, and only 23 (9.8%) physicians recommend a high level of protein, which is recommended for patients on dialysis. Among patients with diabetes with proteinuria, for non-pregnant women, 192 physicians prescribe an ACE inhibitor, which is good practice and recommended for patients with proteinuria to prevent the progression of diabetic kidney disease.

The third part of the questionnaire assessed the practice of physicians in terms of referral. Among patients with diabetes with GFR < 30 mL/min/1.73 on two consecutive visits, 189 physicians refer the patient to a nephrologist, which is good practice and recommended for stage IV kidney disease.

Table 5 displays the factors associated with the level of adherence to clinical guidelines pertaining to the management of patients with or at risk of diabetic nephropathy among the recruited physicians. The adherence level in practice ranged from 40 (higher adherence of the participants) to 19 (lower adherence of the participants), with a median of 33; above 33 was considered a high level of adherence, and lower than 33 was considered a low level of adherence. Higher adherence levels were noted among Saudi Arabian physicians, physicians with high education levels, physicians specializing as family physicians or diabetologists, physicians who reported attending online and on-site training, and those who trained at a diabetes center (*p* < 0.05). Physicians who reported continuous access to urine analysis tests or serum creatinine tests were more likely to have higher adherence levels but with marginal levels of statistical significance (*p* = 0.059 and 0.051, respectively). Finally, physicians who reported having continuous access to the ADA guidelines were more likely to report higher adherence levels in comparison with other physicians without continuous access to those guidelines (*p* < 0.001).

## 4. Discussion

This investigation was a cross-sectional study that targeted physicians working in the Jazan region of Saudi Arabia to measure their practices in the management of diabetic nephropathy in PHC settings. Among the recruited sample of physicians, higher levels of adherence to the guidelines pertaining to the management of diabetic nephropathy were associated with the physicians’ education level, specialty, access to the relevant clinical guidelines, and receipt of relevant training. The availability of the infrastructure and services required for the management of patients at risk of or diagnosed with diabetic nephropathy was associated with the adherence level but at a smaller magnitude compared with the other measured factors.

Evidence concerning the adherence of primary healthcare physicians toward diabetic guidelines regarding the management of patients affected by or at risk of diabetic nephropathy in Saudi Arabia is lacking. Nonetheless, the findings of this investigation can be compared with those of similar international investigations. Physicians’ practices in diabetic nephropathy can be affected by multiple factors related to the training of physicians working in primary healthcare. This study found that physicians who had received training had high adherence to the guidelines on practice in diabetic nephropathy; this finding agrees with that of a similar cross-sectional study conducted among general physicians between 1 March 2015 and 30 September 2015, in Cotonou; it found a positive impact of continuing medical training focusing on attitudes and practices in diabetic nephropathy [12].

Our study found that resources include limited access to diabetic centers, consultation services, nutritionists, health coaches, and ADA guidelines; this finding agrees with that of a qualitative study involving five teaching hospitals in Iran, which found that inadequate physical resources negatively affected nurses’ quality of practice [13]. Also in agreement with this study, it was found that higher adherence is associated with the availability of resources, such as access to the ADA guidelines, and a slightly significant association was found regarding the presence of lab tests for kidney function. A specialty higher than a general practitioner was associated with high adherence to the diabetic nephropathy guidelines. The present study agreed with the finding of another study that good skills and excellence in practice increase with continued medical education and higher degrees of knowledge and specialty [14].

The majority of the physicians recruited in this study showed optimum adherence to the guidelines concerning the recommended interval for the routine screening of diabetic nephropathy. In a study published in 2014 that involved 54,670 patients with type 2 diabetes in Saudi Arabia, it was noted that the overall prevalence of diabetic nephropathy was 10.8%, indicating that the detected prevalence of diabetic nephropathy suggests a need for a screening program in the country [15]. Nonetheless, screening programs for diabetes and similar chronic non-communicable diseases in Saudi Arabia are currently lacking [16].

This study has multiple areas of strengths and weaknesses. The main strength of this study is related to its ability to reach a sample of primary healthcare physicians who are working in different sectors at a regional level, and to assess their adherence to established clinical guidelines in diabetic nephropathy management according to different demographic and working characteristics. The main weakness of this study is related to its dependence on reporting of the recruited physicians, especially concerning the availability of infrastructure pertaining to diabetic nephropathy management, which may subject the findings to measurement bias. Nonetheless, the findings of this investigation are similar to other studies conducted in national and international contexts, and it could be argued that this study has reasonable validity and generalizability to similar clinical settings. The findings of this study indicate the need to strengthen the role of continuous professional development to enhance the quality of care provided by primary healthcare physicians to patients at risk or suffering from diabetic nephropathy. Furthermore, the findings indicate the need to perform subsequent interventional studies to provide evidence concerning best-needed training approaches and educational methods to enhance the quality of care and the adherence level of physicians concerning the management of diabetic nephropathy guidelines.

## 5. Conclusions

Our paper presents findings concerning the degree of adherence to the established guidelines concerning diabetic nephropathy management in real-life clinical settings in a country that has a high prevalence of diabetes and, subsequently, a high risk of development of diabetic nephropathy. This study identified several factors associated with the level of adherence to the guidelines pertaining to the management of diabetic nephropathy, including physicians’ education level, specialty, access to the relevant clinical guidelines, and receipt of relevant training. The findings of this investigation suggest the need to provide more training for PHC physicians in the care of patients affected by or at risk of diabetes. Furthermore, enhanced access to the relevant guidelines is required for all physicians working in PHC settings. Additionally, this study emphasizes the importance of establishing screening for diabetic nephropathy as an innovative indicator to enable appropriate management of patients with diabetes and prevention of the development of complications such as diabetic nephropathy.

## Figures and Tables

**Table 1:** Sociodemographic characteristics of 234 physicians working at primary healthcare centers in Jazan, Saudi Arabia.

Variable	Frequency (Proportion)
Gender	
Male	138 (59%)
Female	96 (41%)
Nationality	
Saudi Arabian	135 (57.7%)
Non-Saudi Arabian	99 (42.3%)
Language	
Arabic	202 (86.3%)
Non-Arabic Speaker	32 (13.7%)
Specialty	
General practitioner	122 (52.1%)
Family physician	111 (47.4%)
Diabetologist *	1 (0.4%)
Education level	
Bachelor degree	123 (52.6%)
High diploma	9 (3.8%)
Master	17 (7.3%)
Board	77 (32.9%)
PhD	3 (1.3%)
Fellowship	5 (2.1%)
Health sector	
Central	45 (19.2%)
Middle	38 (16.2%)
Western	32 (13.7%)
Northern	21 (9%)
Southern	45 (19.2%)
Jabali	25 (10.7%)
Bani Malik	23 (9.8%)
Farasan	5 (2.1%)

* A diabetologist is a physician who is a holder of a postgraduate degree in internal medicine or family medicine and completed a fellowship program in diabetes management.

**Table 2:** Receipt of training concerning diabetic nephropathy among 234 physicians working at primary healthcare centers in Jazan, Saudi Arabia.

Type of Training	Frequency (Proportion)
Receipt of training in diabetes management	
Yes, on-site	34 (14.5%)
Yes, online	47 (20.1%)
Yes, both on-site and online	118 (50.4%)
None	35 (15%)
Receipt of clinical training at a diabetic center	70 (29.9%)
Receipt of clinical training in nephrology	23 (9.8%)
Receipt of practical training on GFR measurement	181 (77.4%)

GFR: glomerular filtration rate.

**Table 3:** Availability of services and resources in primary healthcare centers concerning management of diabetic nephropathy as reported by 234 physicians working at primary healthcare centers in Jazan, Saudi Arabia.

Availability of Resources: Frequency (Proportion)	Always	Sometimes	Never
HGA1c test	96 (41%)	132 (56.4%)	6 (2.6%)
Urine analysis test (proteinuria)	164 (70.1%)	60 (25.6%)	10 (4.3%)
Serum creatinine level test	103 (44%)	92 (39.3%)	39 (16.7%)
Access to Wasfaty services *	200 (85.5%)	27 (11.5%)	7 (3%)
Access to consultation services	51 (21.8%)	114 (48.7%)	69 (29.5%)
Access to a nutritionist	44 (18.8%)	98 (41.9%)	92 (39.3%)
Access to a health coach	72 (30.8%)	93 (39.7%)	69 (29.5%)
Access to ADA guidelines	133 (56.8%)	54 (23.1%)	47 (20.1%)
Access to KDIGO guidelines	35 (15%)	79 (33.8%)	120 (51.3%)

* Wasfaty is an electronic medical service that is provided by the Saudi Arabia Ministry of Health to facilitate prescribing medication via a paperless format.

**Table 4:** Reported practices among 234 physicians working at primary healthcare centers in Jazan, Saudi Arabia, concerning the care of patients with or at risk of diabetic nephropathy.

Statement	Frequency [Proportion]
The frequency of performing routine screening for diabetic nephropathy is:	
Three times/year	9 (3.8%)
Twice/year	44 (18.8%)
Annually *	181 (77.4%)
Initiation of screening of diabetic nephropathy in type 1 DM is:	
Initially at diagnosis	81 (34.6%)
1 year after diagnosis	31 (13.2%)
5 years after diagnosis *	122 (52.1%)
Initiation of screening of diabetic nephropathy in type 2 DM is:	
5 years after diagnosis	25 (10.7%)
1 year after diagnosis	32 (13.7%)
Initially at diagnosis *	177 (75.6%)
The utilized method for screening diabetic nephropathy is:	
Renal ultrasound	6 (2.6%)
HGA1c	28 (12%)
Albumin/creatinine ratio *	200 (85.5%)
Frequency of follow-up visits among patients with urinary albumin = 300 mg/g creatinine and GFR ≥ 90 mL/min/1.73:	
Annually	98 (41.9%)
Three times per year	85 (36.3%)
Twice annually *	51 (21.8%)
Frequency of follow-up visits among patients with urinary albumin = 300 mg/g creatinine and GFR = 30–60 mL/min/1.73:	
Annually	29 (12.4%)
Three times per year	105 (44.9%)
Twice annually *	100 (42.7%)
Utilized medication for patients diagnosed with type 2 diabetes, chronic kidney disease eGFR > 25 mL/min/1.37, and urinary albumin = 300 mg/g creatinine:	
Metformin	17 (7.3%)
Sitagliptin	44 (18.8%)
Empagliflozin *	173 (73.9%)
Utilized medication for patients diagnosed with type 2 diabetes, chronic kidney disease, risk of cardiovascular disease, and contraindication to use an SGLT2 inhibitor:	
Captopril	85 (36.3%)
Valsartan	88 (37.6%)
Finerenone *	61 (26.1%)
Practices concerning a patient who started on ACEI/ARBs, with serum creatinine increasing by <30% without volume depletion:	
To stop medication and change to another class	79 (33.8%)
To stop without adding another class	14 (6%)
To continue the same medication *	141 (60.3%)
The prescribed amount of protein prescribed for diabetic kidney disease patients on dialysis is:	
0.7 g/kg of body weight/day	41 (17.5%)
0.6 g/kg of body weight/day	139 (59.4%)
0.8 g /kg of body weight/day *	54 (23.1%)
Medication prescribed for diabetic, non-pregnant women, with proteinuria:	
Thiazide diuretic	18 (7.7%)
Beta blocker	24 (10.3%)
ACE inhibitor *	192 (82.1%)
The level of protein intake advised for patients with diabetes and stage 5 chronic kidney disease on dialysis is:	
Low level of protein	166 (70.9%)
Medium level of protein	45 (19.2%)
High level of protein *	23 (9.8%)
Practices concerning diabetic nephropathy patients with eGFR < 30 mL/min/1.73 on two consecutive visits are to:	
Schedule one visit/year to measure GFR	19 (8.1%)
Consult internal medicine without a referral	26 (11.1%)
Refer to a nephrologist *	189 (80.8%)
Practices concerning a patient with rapid progressive kidney disease are:	
Follow up in PHC without a referral	8 (3.4%)
Consult internal medicine without a referral	22 (9.4%)
Refer to a nephrologist *	204 (87.2%)

* Adequate practices.

**Table 5:** Factors associated with the practices of 234 physicians working at primary healthcare centers in Jazan, Saudi Arabia, concerning the care of patients with or at risk of diabetic nephropathy.

Measured Characteristics	Practice Adequacy: Frequency (Proportions)		*p* Value
	High Adherence	Low Adherence	
Gender *			0.421
Male	85 (61.2%)	53 (38.4%)	
Female	54 (56.2%)	42 (43.8%)	
Nationality *			0.022
Saudi Arabian	89 (65.9%)	46 (34.1%)	
Non-Saudi Arabian	50 (50.5%)	49 (49.5%)	
Language *			0.252
Arabic	123 (60.9%)	79 (39.1%)	
Non-Arabic speaker	16 (50%)	16 (50%)	
Education *			<0.001
Bachelor, high diploma, master	73 (49%)	76 (51%)	
Board, PHD, fellowship	66 (77.6%)	19 (22.4%)	
Specialty *			<0.001
GP	52 (42.6%)	70 (57.4%)	
Family medicine or diabetologist	87 (77.7%)	25 (22.3%)	
Receipt of training in diabetes management *			0.011
Yes, on-site	19 (55.9%)	15 (44.1%)	
Yes, online	22 (46.8%)	25 (53.2%)	
Yes, both online and on-site	82 (69.5%)	36 (30.5%)	
None	16 (45.7%)	19 (54.3%)	
Receipt of clinical training at a diabetic center *			0.020
Yes	50 (71.4%)	20 (28.6%)	
No	89 (54.3%)	75 (45.7%)	
Receipt of clinical training in nephrology *			0.506
Yes	12 (52.2%)	11 (47.8%)	
No	127 (60.2%)	84 (39.8%)	
Receipt of practical training on GFR measurement *			0.025
Yes	115 (63.5%)	66 (36.5%)	
No	24 (45.3%)	29 (54.7%)	
Availability of HGA1c test **			0.009
Always	57 (59.4%)	39 (40.6%)	
Sometimes	82 (62.1%)	56 (37.9%)	
Never	0 (0%)	6 (100%)	
Availability of urine analysis test **			0.061
Always	104 (63.4%)	60 (36.6%)	
Sometimes	32 (53.3%)	28 (46.7%)	
Never	3 (30%)	7 (70%)	
Availability of Serum creatinine level *			0.051
Always	68 (66%)	35 (34%)	
Sometimes	54 (58.7%)	38 (41.3%)	
Never	17 (43.6%)	22 (56.4%)	
Access to Wasfaty services **			0.229
Always	123 (61.5%)	77 (38.5%)	
Sometimes	12 (44.4%)	15 (55.6%)	
Never	4 (57.1%)	3 (42.9%)	
Access to consultation services *			0.299
Always	35 (68.5%)	16 (31.4%)	
Sometimes	62 (54.4%)	52 (54.6%)	
Never	42 (60.9%)	27 (39.1%)	
Access to nutritionist *			0.444
Always	27 (61.4%)	17 (38.6%)	
Sometimes	62 (63%)	36 (36.7%)	
Never	50 (54.3%)	42 (45.7%)	
Access to a health coach *			0.597
Always	41 (56.9%)	31 (43.1%)	
Sometimes	59 (63.4%)	34 (36.6%)	
Never	39 (56.5%)	30 (43.5%)	
Access to KDIGO guidelines *			0.426
Always	24 (68.6%)	11 (31.4%)	
Sometimes	44 (55.7%)	35 (44.3%)	
Never	71 (59.2%)	49 (40.8%)	
Access to ADA guidelines *			<0.001
Always	96 (72.2%)	37 (27.8%)	
Sometimes	21 (38.9%)	33 (61.1%)	
Never	22 (46.8%)	25 (53.2%)	

* Chi-Square test. ** Fisher’s Exact test.

## Data Availability

The data presented in this study are available upon request from the corresponding author.
